# The monkeypox virus suppresses autophagy by modulating Rubicon expression

**DOI:** 10.1038/s41420-025-02920-z

**Published:** 2025-12-23

**Authors:** Giulia Refolo, Cosmina Mija, Fabiola Ciccosanti, Giuseppe Sberna, Valentina Mazzotta, Fabrizio Maggi, Mauro Piacentini, Tiziana Vescovo, Licia Bordi

**Affiliations:** 1 Laboratory of Cellular Biology and Electron Microscopy, National Institute for Infectious Diseases “Lazzaro Spallanzani” -IRCCS, Rome, Italy; 2 Laboratory of Virology and Laboratories of Biosafety, National Institute for Infectious Diseases “Lazzaro Spallanzani” -IRCCS, Rome, Italy; 3 Counseling, Test and HIV Prophylaxis and STI Unit, Regional AIDS Reference Center, National Institute for Infectious Diseases “Lazzaro Spallanzani”- IRCCS, Rome, Italy; 4 Department of Biology, University of Rome “Tor Vergata”, Rome, Italy

**Keywords:** Viral infection, Macroautophagy

## Abstract

Monkeypox virus (MPXV) is a globally reemerging pathogen that poses a significant threat to public health, representing the most impactful Orthopoxvirus infection in humans since the eradication of smallpox. Macroautophagy (hereafter referred to as autophagy) is an evolutionarily conserved catabolic process essential for maintaining cellular homeostasis, and it can exert either pro-viral or anti-viral effects during infections. Poxviruses interaction with the autophagy machinery remains poorly understood, and the specific interplay between MPXV and autophagy has not been documented. In this study, we infected Calu-3 cells with MPXV and observed that the virus significantly impairs autophagic flux by upregulating Rubicon, a known negative regulator of autophagy. Notably, silencing Rubicon restored autophagic flux and led to a marked reduction in MPXV replication. Overall, our findings reveal a novel mechanism by which MPXV inhibits autophagy through the modulation of Rubicon, suggesting that autophagy activation may be a potential therapeutic strategy for MPXV.

## Introduction

The monkeypox virus (MPXV) is a double-stranded DNA virus of ~197.2 kilobases belonging to the *Orthopoxvirus* genus, subfamily Chordopoxvirinae, family Poxviridae [1], which has a strong impact on the public health system, being the causative agent of monkeypox (Mpox), a zoonotic disease of international concern [1–3]. The virus is divided into two clades: Clade I (also referred to as the Central African clade) and Clade II (also referred to as the West African clade), both presenting subclades *a* and *b* [1]. In addition to animal-to-human-transmission, with rodents being identified as primary MPXV reservoirs, human-to-human-transmission occurs following close physical contact with lesions or body fluids or through respiratory secretions of infected persons [2, 4]. The rate of transmission, as well as the severity of symptoms, is clade-related, with Clade Ib being less pathogenic but more easily transmissible than Clade IIb [5], which explains the rapid spread of Mpox disease during the 2024 outbreak.

The disease is usually mild, starting with nonspecific symptoms (fever, chills, headaches, muscle aches, and back pain) and subsequently progressing to the enlargement of lymph nodes and the appearance of skin lesions, two typical manifestations of Mpox disease [2]. Although the majority of cases in humans present a self-limiting course, complications have been reported, especially in immunocompromised subjects infected with Clade IIb, related to the involvement of the respiratory system [6, 7]. Indeed, patients infected with MPXV are known to experience respiratory symptoms, such as coughing, dyspnea, nasal congestion, and secondary bacterial infections, which can lead to severe complications, including bronchopneumonia and pharyngeal inflammation [8]. Analyses performed on genomes belonging to the B.1 lineages suggest that the virus harbors multiple mutations in genes with functional implications in infection and transmission, thus contributing to its adaptability, pathogenicity, virulence, transmissibility, and immune evasion, favoring the rapid spread of MPXV into non-endemic countries [9].

A peculiar feature of MPXV and all poxviruses is that the life cycle is exclusively cytosolic; indeed, after the viral entry into the host cell, the genome is released, and viral replication occurs in the cytoplasm of infected cells with a hierarchical transcription of early, intermediate, and late genes [10]. This unique characteristic requires MPXV to hijack and manipulate various cellular processes and machinery to successfully replicate, encoding proteins that interfere with host cell defenses, redirect cellular resources, and create specialized replication factories within the cytoplasm. Therefore, there is an urgent need to better understand the virus–host interplay, to identify new potential targets for antiviral interventions, and to improve global health responses to MPXV.

Autophagy is a self-degradative process crucial for cellular homeostasis, balancing energy sources at critical times during development and in response to nutrient stress. Autophagy is a multistep process whose initiation is under the control of the nutrient sensor mTOR (serine/threonine kinase mammalian target of rapamycin). Under normal conditions, mTOR represses autophagy induction, while nutrient starvation conditions or treatment with mTOR inhibitors [11, 12], lead to autophagy initiation and nucleation of a phagophore membrane, likely derived from lipid bilayer contributed by the endoplasmic reticulum and/or the trans-Golgi and endosomes [13, 14]. This phagophore expands in a double-membraned autophagosome (AP) to engulf intracellular cargo, such as protein aggregates or organelles. The loaded AP matures through fusion with the lysosome, promoting the degradation of autophagosomal contents by lysosomal acid proteases [15].

Each step of the autophagy process is under the control of several autophagy-regulating proteins; among them, the RUN domain Beclin-1-interacting cysteine-rich-containing (Rubicon) protein, first identified as a Beclin-1-binding partner, localizes to the late endosome/lysosome and negatively regulates the maturation step of autophagy, but it is now evident that it can also modulate autophagy initiation [16, 17]. Notably, Rubicon, binding to the PI3K–UVRAG complex, suppresses autophagy initiation by inhibiting PI3K activity [18], while interacting with UVRAG, inhibits APs fusion with lysosomes, thus impairing APs maturation and degradation [16, 17]. In addition, Rubicon suppresses autophagy and endosomal trafficking by directly interacting with Rab7 [19].

Depending on the cargos being sorted for destruction, autophagy can be classified into several selective forms. Specifically, xenophagy is a type of selective autophagy that senses intracellular microorganisms, including viruses, which are recognized by a subclass of autophagy receptors for pathogen detection, namely, the SQSTM1/p62 (Sequestosome-1)-like receptors: SQSTM1/p62, NBR1, NDP52, Tax1Bp1, and Optineurin (OPTN) [20]. In response to viral infections, autophagy can be activated to promote an antiviral state within the infected cell, either by selectively directing viral particles to the lysosome for degradation (virophagy), stimulating IFN production by activating the host’s innate immune response, or by coordinating adaptive immunity through the promotion of viral antigen presentation [21]. Although autophagy aims at clearance, some viruses have evolved a variety of strategies to inhibit, escape, manipulate, and subvert one or multiple steps of autophagy to the elemental goal of survival, replication, and propagation. Physically, the double-membrane compartments, formed during autophagy, can provide protection for viral components from immune surveillance, and metabolically, viruses can utilize autophagy-generated energy and metabolites [21]. On the other hand, viruses may suppress the autophagic process to avoid degradation or interfere with APs maturation in order to accumulate and exploit autophagosomal membranes as a platform for viral replication or assembly [22]. Although the involvement of autophagy in certain viral infections is well characterized, the role of autophagy in poxvirus infections and the strategies employed by poxviruses to manipulate autophagy have not been extensively explored and remain controversial.

To date, there is no evidence in the literature describing a possible interplay between MPXV infection and autophagy. Considering that severe respiratory complications may occur during the Mpox disease, this study investigated a possible involvement of the autophagic process in the course of MPXV infection in a human bronchial adenocarcinoma cell line (Calu-3 cells). Our results indicate that MPXV inhibits autophagic flux to ensure its replication by regulating the levels of Rubicon.

## Results and discussion

### Analysis of autophagic flux in Calu-3 cells infected with MPXV

To investigate a potential role of MPXV infection in the modulation of the autophagic process, Calu-3 cells were infected with MPXV at a Multiplicity of Infection (MOI) of 0.5 and 1 for 24 and 48 h.

**Fig. 1:**
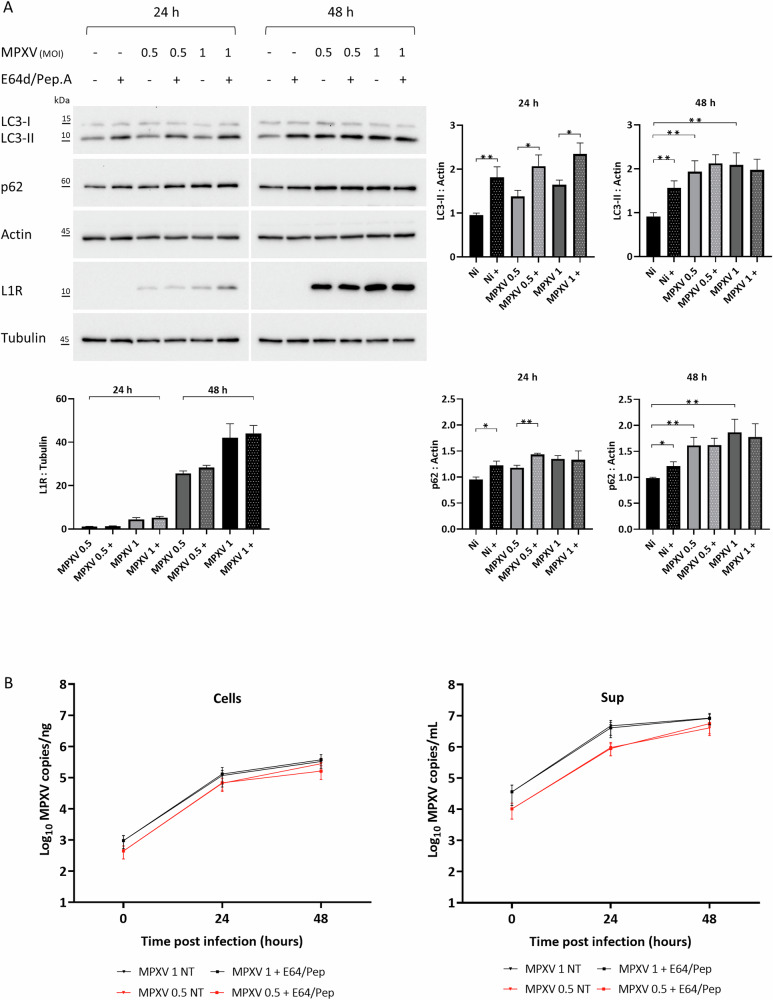
MPXV infection impairs autophagy flux. **A** Calu-3 cells were infected with MPXV at an MOI of 0.5 or 1 for 24 and 48 h. Two hours before lysis, cells were treated with the lysosome inhibitors E64d/Pep.A as indicated (+). LC3-II and p62 levels were analyzed to monitor autophagy flux, while L1R levels were analyzed to control MPXV replication by western blot. Actin and Tubulin were included as a loading control. The graphs represent mean ± SEM of LC3-II:Actin, p62:Actin and L1R:Tubulin values from at least three independent experiments. **B** Calu-3 cells were infected with MPXV at MOI of 0.5 (represented in red) or 1 (represented in black) for 24, 48 h, and untreated (NT) or treated with E64d/Pep.A for 2 h. Kinetic of viral yield inside the cells (Cells) (expressed as Log copies/ng) and in supernatants (Sup) (expressed as Log copies/mL) were quantified by qRT-PCR. Experiments were performed as three independent replicates; mean ± SEM are shown in the picture.

In MPXV-infected cells, autophagy flux activity was monitored by detection of the autophagosomal marker LC3-II (microtubule-associated proteins 1 light chain 3) protein levels in the presence or absence of lysosomal inhibitors through western blot analysis (Fig. 1A). Accordingly, two hours before lysis, cells were treated with E64d and Pepstatin A (E64d/Pep.A), which impair lysosome degradative activity and consequently block autophagy at late stages, leading to immature APs accumulation [23].

As shown in Fig. 1A, at 24 hours (h) post-infection (p.i.), no perturbation of autophagic flux was observed in MPXV-infected cells with respect to uninfected ones since LC3-II levels were comparable and increased following E64d/Pep.A treatment, thus indicating the presence of an active and functional autophagic flux. In contrast, at 48 h, our results showed that LC3-II protein accumulated after MPXV infection compared to mock-infected Calu-3 cells, thus suggesting that MPXV may promote APs formation or impair autophagic degradation. Considering that the use of E64d/Pep.A did not lead to a further increase of LC3-II levels in MPXV-infected cells, the observed APs accumulation, at 48 h p.i., may be the result of autophagic degradation inhibition caused by MPXV infection (Fig. 1A).

Consistently, the levels of the autophagic receptor SQSTM1/p62 also increased at 48 h p.i. in MPXV-infected cells compared to uninfected (Fig. 1A), supporting that MPXV impairs autophagy at late stages.

MPXV infection was assessed by western blot detection of the viral protein L1R (Fig. 1A) and by viral DNA quantification (Fig. 1B). Specifically, MPXV replication kinetics denoted that E64d/Pep.A treatment did not affect viral replication, since no significant differences in MPXV genome copies number have been observed either in supernatants (Sup) or at the intracellular level (Cells) (Fig. 1B). Notably, our results revealed a novel interplay between MPXV and autophagy, providing evidence on the MPXV ability to inhibit autophagy degradation during its infection, thus resulting in the accumulation of immature APs.

It is well-documented that several viruses impair autophagy at late stages to avoid their autophagic degradation or to accumulate autophagosomal membranes, exploited as platforms for viral replication [21]. To investigate whether APs accumulation is required for an efficient MPXV replication, we inhibited early stages of autophagy by silencing the expression of the ATG7 gene (E1-like ubiquitin-activating enzyme autophagy-related gene 7). ATG7 is a crucial protein involved in autophagy by promoting the formation and extension of autophagosome membranes [24]. ATG7 expression was downregulated by RNA Interference in Calu-3 cells using two different ATG7-specific oligonucleotides (iATG7#1, iATG7#2), and a nonspecific oligonucleotide (iCtr), as a control. Cells were then infected with MPXV at an MOI of 0.5 for 48 h. The efficiency of ATG7 downregulation was verified by western blot (Fig. S1B). In Fig. S1A, as previously reported on Fig. 1A, in iCtr cells infected with MPXV, we observed a significant accumulation of APs, which did not further increase following E64d/Pep.A treatment, confirming that MPXV blocks autophagic flux. As expected, ATG7 silencing (iATG7#1 and iATG7#2) caused a reduction in LC3-II levels both in E64d/Pep.A treated and untreated condition, confirming an impairment in autophagy and APs formation.

Further, we investigated ATG7 depletion effects on MPXV replication. ATG7 downregulation caused a mild decrease in viral protein L1R (Fig. S1C) and a reduction of both viral replication and production of new viral particles (Fig. S1D) compared to the control, especially at 48 h p.i. Viral infectivity assessed at 48 h p.i. in supernatants further supports the previous results: the MPXV titer is significantly decreased in iATG7#1 and iATG7#2 cells with respect to iCtr cells (Fig. S1E).

Collectively, our results indicate that AP formation is relevant for MPXV replication, thus AP accumulation, reported at 48 h p.i. (Fig. 1A), could assist the MPXV life cycle.

### Analysis of Rubicon protein levels in MPXV-infected cells

**Fig. 2:**
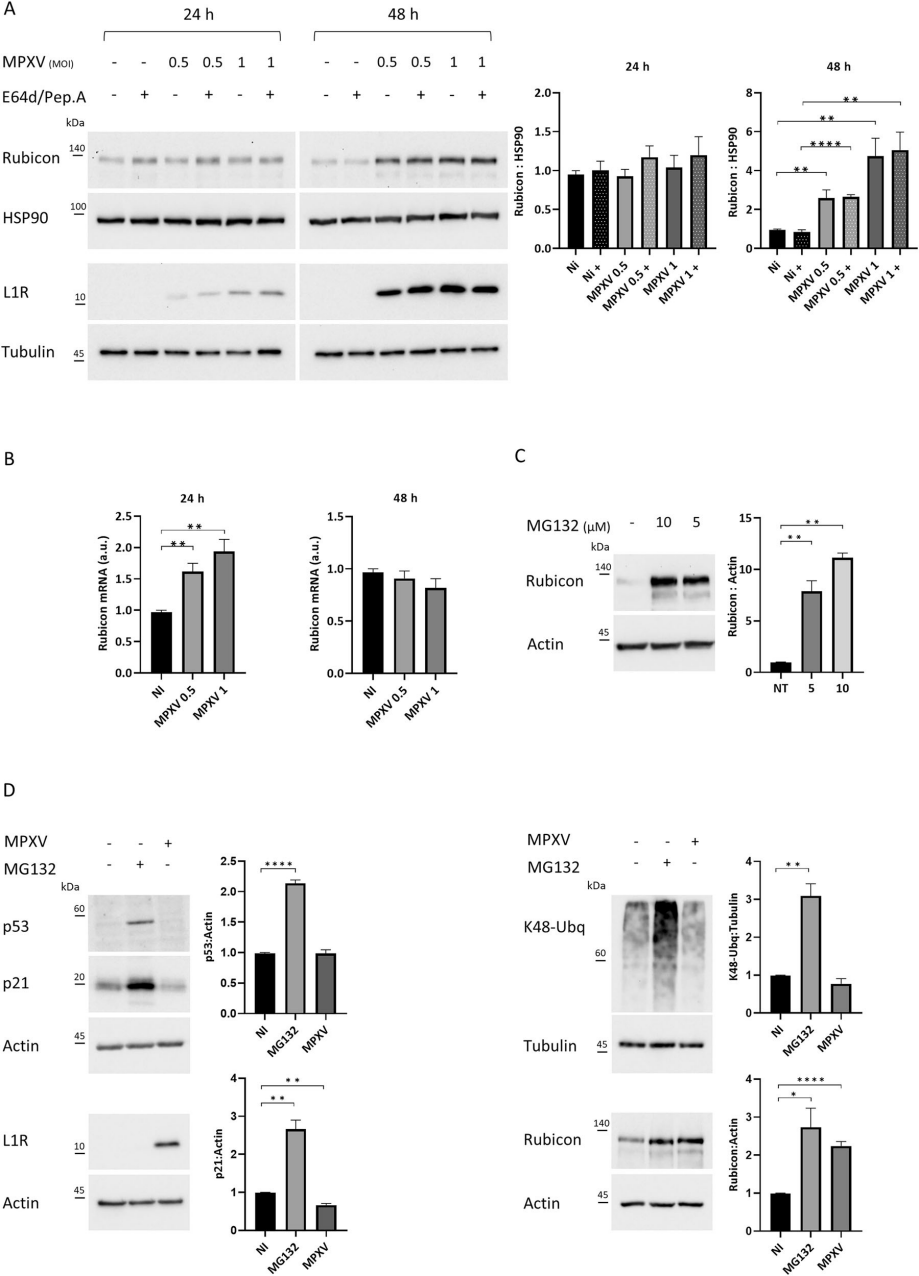
MPXV infection increases Rubicon levels. **A** Calu-3 cells were infected with MPXV at an MOI of 0.5 or 1 for 24 h and 48 h. Two hours before lysis, cells were incubated with E64d/Pep.A as indicated (+). Rubicon and L1R levels were analyzed by western blot. HSP90 and Tubulin were included as loading controls. The graphs represent mean ± SEM of Rubicon:HSP90 values from three independent experiments. **B** Real-time PCR analysis of Rubicon mRNA levels in Calu-3 cells infected with MPXV at MOI of 0.5 or 1 for 24 or 48 h. Expression levels were normalized based on HSP90 values. A.U. arbitrary unit. Graph reports mean ± SEM of values from 3 independent experiments. **C** Calu-3 cells were treated for 4 h with the proteasomal inhibitor MG132 at a final concentration of 10 or 5 µM. Rubicon levels were analyzed by western blot. Actin was included as a loading control. The graphs represent mean ± SEM of Rubicon:Actin values from three independent experiments. **D** Calu-3 cells were treated for 4 h with the proteasomal inhibitor MG132 at a final concentration of 5 µM or infected with MPXV at MOI of 0.5 48 h. p21, p53, K48-linked ubiquitin (ubq) chains, and Rubicon levels were analyzed by western blot. L1R levels were monitored to verify MPXV replication. Actin and Tubulin were included as loading controls. The graphs represent mean ± SEM of p21:Actin, p53:Actin, L1R:Actin K48-Ubiquitin:Tubulin, Rubicon:Actin values from three independent experiments.

To investigate the mechanism by which MPXV impairs autophagy, we evaluated whether the virus is able to modulate proteins involved in the regulation of autophagy by western blot analysis. Among all the screened autophagic regulators and receptors (Fig. S2), we found the levels of Rubicon (RUBCN) significantly increased (up to five-fold) after 48 h of MPXV infection (Fig. 2A). It is well known that Rubicon acts as a negative regulator of autophagy by inhibiting autophagosome maturation, and notably, autophagy inhibition performed by MPXV occurred at 48 h p.i. (Fig. 1), exactly when the increase of Rubicon was observed, suggesting that the virus may use Rubicon activity to perturb the autophagic process. To investigate whether the MPXV-induced Rubicon increase may be due to a transcriptional up-regulation, Rubicon gene expression levels were analyzed by quantitative PCR in Calu-3 cells infected with MPXV at MOI of 0.5 and 1 for 48 h. The results showed that Rubicon gene expression mildly increased in MPXV-infected cells proportionally to the MOI (Fig. 2B). Considering that Rubicon up-regulation in gene expression (up to 2-fold) only partially correlates with the high increase in Rubicon protein levels (up to 5-fold) observed by western blot (Fig. 2A), it could be reasonable that Rubicon production is being regulated mostly at the post-transcriptional (e.g., translation or protein degradation) rather than at the transcriptional level.

In this regard, we evaluated Rubicon proteasomal degradation in Calu-3 cells. To this aim, cells were treated for 4 h with increasing concentrations (5 and 10 µM) of MG132, a potent proteasome inhibitor. Figure 2C shows a large, dose-dependent accumulation of Rubicon protein, indicating that naïve Calu-3 cells undergo a high proteasomal turnover of Rubicon. Based on this evidence, it can be speculated that during MPXV infection, Rubicon is being degraded at a slower rate than usual, suggesting that MPXV may interfere with the ubiquitin-proteasome system in order to accumulate Rubicon protein.

In order to clarify whether MPXV inhibits proteasomal activity or acts specifically on Rubicon stability, we tested the levels of p21 and p53, known proteasomal substrates that usually undergo a rapid turnover by the proteasome [25, 26]. In particular, Calu-3 cells were infected with MPXV at an MOI of 0.5 for 48 h or treated with 5 µM MG132 for 4 h, and the levels of p21, p53 were analyzed by western blot. As reported in Fig. 2D, p21 and p53 protein levels, which strongly accumulate upon MG132 treatment, result unaltered following MPXV infection. Moreover, we monitored the amount of K48-linked ubiquitin chains, which reflects the levels of proteasomal activity [27], and consistently with previous results, no alterations were observed following MPXV infection. All these data suggest that MPXV specifically inhibits Rubicon proteasomal degradation by increasing its stability rather than indiscriminately impairing proteasomal activity.

Notably, recent studies have demonstrated that Rubicon plays a strategic role in viral replication. In this regard, several viruses increase Rubicon levels to suppress the autophagy pathway and escape the autophagic degradation or to inhibit the activity of essential effectors of cGAS-STING/TBK1, thus perturbing the antiviral IFN response and favoring viral immune escape [28].

As reported in the literature, during viral replication, Rubicon upregulation allows the virus to spread, while inhibition of Rubicon might be beneficial for the prevention of viral infection, thus highlighting Rubicon as a new candidate for developing new therapeutic strategies for various infectious diseases.

### Effects of Rubicon gene silencing on autophagic flux in MPXV-infected cells

**Fig. 3:**
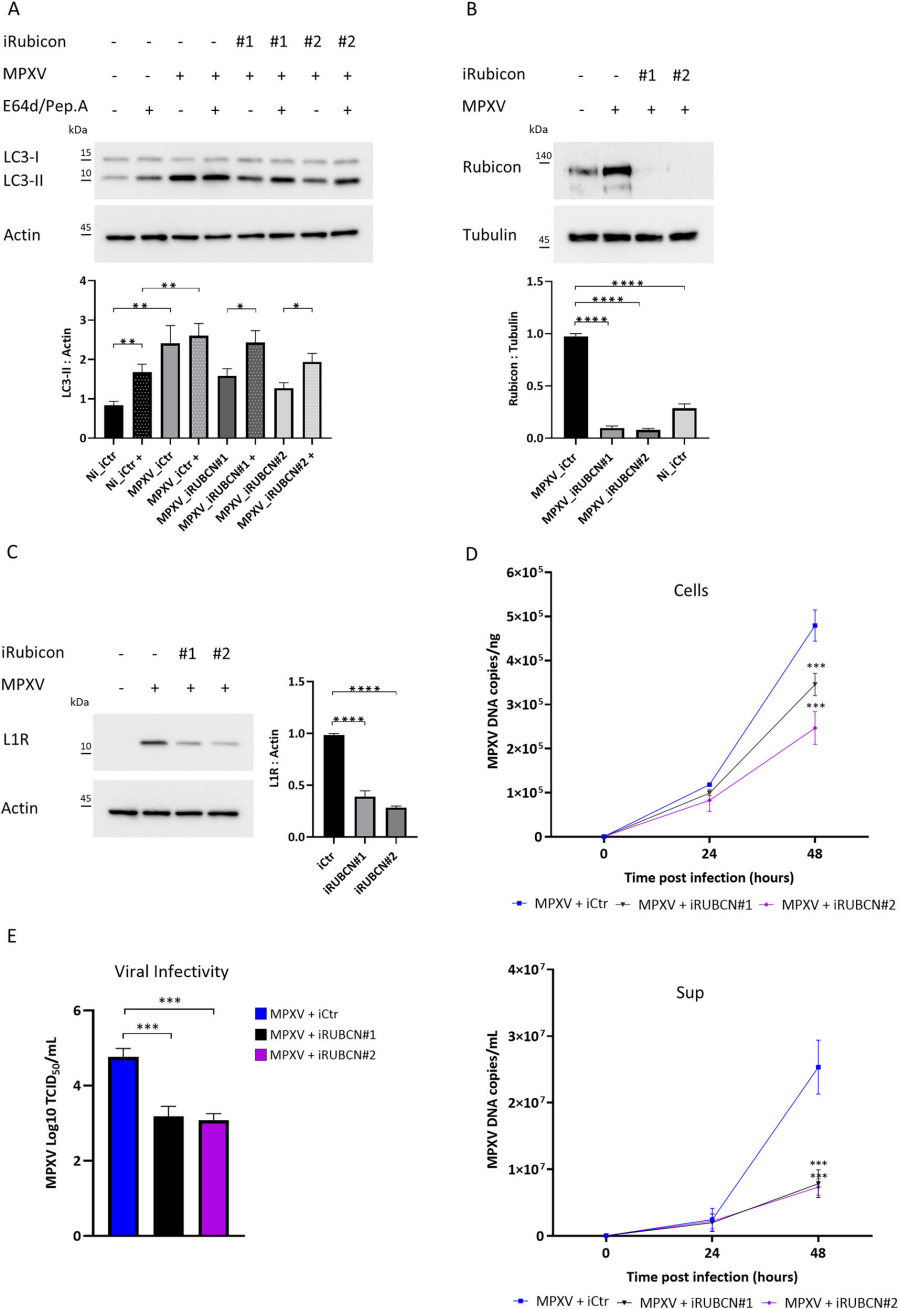
Rubicon depletion restores autophagy flux and impairs MPXV replication. Calu-3 cells silenced for Rubicon gene expression (iRUBCN#1, iRUBCN#2) or not (iCtr), were infected at an MOI of 0.5 and cultured for 48 h. Two hours before lysis, cells were incubated with E64d/Pep.A as indicated. **A** LC3-II levels were analyzed to monitor autophagy flux by western blot. Actin was included as a loading control. The graph represents mean ± SEM of LC3-II:Actin values from three independent experiments. **B** Rubicon levels were analyzed by western blot. Tubulin was included as a loading control. The graphs represent means ± SEM of Rubicon:Tubulin values from three independent experiments. **C** L1R levels were analyzed to verify MPXV replication by western blot. Actin was included as a loading control. The graphs represent mean ± SEM of L1R:Actin values from three independent experiments. **D** Calu-3 cells were silenced for Rubicon gene expression: iRUBCN#1 (represented in black), iRUBCN#2 (represented in purple), or not (iCtr, represented in blue), infected at an MOI of 0.5 and cultured for 48 h. Kinetics of viral yield inside the cells (Cells) (expressed as copies/ng) and in supernatants (Sup) (expressed as copies/mL) were quantified by qRT-PCR. **E** Viral titer was evaluated as Log TCID50/mL in supernatants of Calu-3 infected cells with MPXV at MOI of 0.5 for 48 h, and transfected either with iRUBCN#1 (represented in black) or iRUBCN#2 (represented in purple) and iCtr (represented in blue). Experiments were performed as three independent replicates; mean ± SEM are shown in the picture.

To study the role of the Rubicon protein during MPXV infection, its gene was silenced by RNA Interference. Silencing was performed on Calu-3 cells using two different Rubicon-specific oligonucleotides (iRUBCN#1, iRUBCN#2), and a nonspecific oligonucleotide (iCtr), as a control. Cells were then infected with MPXV at an MOI of 0.5 for 48 h. The efficiency of Rubicon downregulation was verified by western blot (Fig. 3B). In Fig. 3A, western blot analysis of LC3-II protein indicated that Rubicon depletion was able to prevent MPXV-induced inhibition of autophagic flux in infected cells. As expected, at 48 h p.i., in iCtr cells infected with MPXV, we observed a significant accumulation of APs, marked by LC3-II, which did not further increase following E64d/Pep.A treatment, confirming the autophagic flux impairment induced by MPXV. In contrast, iRUBCN#1 and iRUBCN#2 cells infected with MPXV presented LC3-II levels comparable to uninfected cells, which increased following E64d/Pep.A treatment, thus demonstrating the presence of a functional autophagic flux and lysosomal turnover. These results confirmed that MPXV, increasing Rubicon levels, utilizes its functions to inhibit autophagy, while Rubicon downregulation restores a functional autophagic flux in MPXV-infected cells. Since Rubicon downregulation caused a significant decrease in viral protein L1R (Fig. 3C), we investigated whether Rubicon depletion and the consequent autophagy restoration could affect viral replication.

To this aim, Calu-3 cells silenced for Rubicon gene expression (iRUBCN#1, iRUBCN#2), or not (iCtr), were infected with MPXV at an MOI of 0.5 for 24 or 48 h, and viral DNA was quantified from cells and from their culture media (Sup). Interestingly, Rubicon downregulation drastically reduced both viral replication and the production of new viral particles, compared to the control, with the greatest differences reported at 48 h p.i. (Fig. 3D), thus confirming that MPXV exploits Rubicon to efficiently suppress autophagy and promote its life cycle.

Viral infectivity has been assessed at 48 h p.i. in supernatants of infected iRUBCN#1 and iRUBCN#2 cells, thus supporting the previous results: the MPXV titer significantly decreased in iRUBCN#1 and iRUBCN#2 cells with respect to iCtr cells, confirming Rubicon’s implication in the MPXV life cycle. These results agree with studies investigating the role of Rubicon in other viral infections.

It has been described that Hepatitis C Virus (HCV) infection suppresses autophagy by increasing Rubicon expression levels in hepatocytes [29]. Notably, HCV promotes its replication by inducing the expression of Rubicon, which leads to the accumulation of immature APs in the early time points of viral infection. In this context, the suppression of Rubicon expression enhances APs maturation and reduces HCV replication [30]. Kaposi’s sarcoma-associated herpesvirus inhibits APs maturation through the interaction between K7 viral protein and Rubicon in order to evade the host defense system via inhibition of autophagy activity [31].

Furthermore, Hepatitis B virus, enterovirus 71, influenza A virus, and vesicular stomatitis virus can upregulate Rubicon, which, in turn, impairs IFN induction and cellular antiviral response, thereby promoting viral replication [28].

Considering that high levels of Rubicon have been found in several biological samples from patients with viral infections [28, 29], it is possible to speculate that Rubicon may represent not only a marker of viral infection, but also a promising therapeutic target. Indeed, the modulation of Rubicon levels would restore autophagy and an effective immune response, improving the prognosis of infected patients. Moreover, a recent multi-omics characterization of MPXV infection reveals that the virus affects host cell metabolism by dysregulating mTOR and the translation machinery, thereby diverting host resources towards the biosynthesis of its own components [32]. In this work, Torin 2, an experimental drug targeting mTOR, successfully inhibits MPXV replication in line with previous reports on the antiviral activity of Torin against the Vaccinia virus [32, 33]. Since Torin 2 also functions as a potent inducer of autophagy [34], it could be speculated that autophagy stimulation may have a detrimental effect on MPXV replication.

Considering the genetic variability of MPXV, which could make it difficult to identify viral proteins as therapeutic targets, the strategy of targeting the host instead of viral factors is arguably less prone to resistant mutations and represents a more promising treatment for MPXV control.

Finally, we reported the first evidence, to our knowledge, of the interplay between MPXV and autophagy. Our results showed that MPXV infection affects autophagic degradation, resulting in the increase of LC3-II and SQSTM/p62 in Calu-3 cells. Notably, AP formation and accumulation play an important role in MPXV infection, since ATG7 depletion affects MPXV replication.

The MPXV-induced negative regulation of autophagy is mediated by Rubicon, whose levels are highly increased during MPXV infection. Rubicon downregulation restores a functional autophagy flux and, restricting the amount of APs, significantly affects MPXV replication in infected cells.

In conclusion, MPXV exploits AP formation, hijacks Rubicon functions to inhibit autophagy at late stages, thereby promoting viral replication and efficiently accomplishing its life cycle.

## Materials and methods

### Cells and virus stock preparation

Calu-3-HTB-55 (lung epithelial adenocarcinoma cells) (ATCC) and Vero E6-CRL-1586 (immortalized African green monkey kidney epithelial cells) (ATCC) were cultured in modified Eagle medium (MEM; Sigma-Aldrich, M2279), supplemented with fetal bovine serum (FBS (Gibco, 10270)) at a final concentration of 10%. Cells were screened for Mycoplasma contamination (PCR Mycoplasma Detection Kit; abmGood, Cat#G238). Cells were grown in a humidified incubator at 37 °C and 5% CO_2_. To block lysosomal activity, cells were treated with E64d and Pepstatin A (5 μg/mL) (Santa Cruz Biotechnology, sc-201280A, sc-45036) for 2 h before lysis.

For the virus stock preparation, Vero E6 cells were infected with the human MPXV 2022 isolate, Italy, strain hMpxV/Italy/un-INMI-Pt2/2022, clade/lineage IIb B.1 (GISAID: EPI_ISL_13251120, GenBank: ON745215.1), obtained from a skin lesion of an MPXV-infected patient. After infection, cells were cultured until the cytopathic effect (CPE) appeared. The supernatant of the infected cells underwent three freezing/thawing cycles, and then it was centrifuged in order to remove cellular debris. Then, it was collected, aliquoted, and stored at −80 °C. To obtain the virus titer, a limiting dilution assay was performed on Vero E6 cells, and results were reported as 50% tissue culture infectious dose per milliliter (TCID_50_/mL). Using the Reed and Muench method [35], the virus stock titer was calculated to be 10^7.25^ TCID_50_/mL, corresponding to 1.2 × 10^7^ plaque-forming units (PFU). All procedures described below involving infectious MPXV were performed in a Biosafety Level 3 (BSL-3) facility, following standard operating procedures approved by the Institutional Biosafety Committee.

#### In-vitro infection experiments

To examine if the infection affects the autophagic flux, Calu-3 cells were plated at 150,000 cells/well in six-well plates, cultured for 24 h at 37 °C and 5% CO_2_, and then infected with the hMpxV/Italy/un-INMI-Pt2/2022 isolate using two different MOI of 0.5 and 1 TCID_50_/cell. Uninfected cells were used as a control. After 1 h and 30 min of incubation, the inoculum was removed, cells were rinsed with warm phosphate-buffered saline (PBS; Corning, 21-031-CV), and grown with medium containing 10% FBS. Both not-infected and infected cells were treated with E64d/Pep.A 2 h before collection. At 0, 24, and 48 h. p.i. supernatants and cells were separately collected for both uninfected treated/not treated and infected treated/not treated conditions.

### RNA interference experiments

To investigate the role of Rubicon or ATG7 during viral replication, silencing of their gene expression has been performed. To this aim, Calu-3 cells were transfected with lipofectamine RNAiMAX (Invitrogen, 13778150) with two specific oligonucleotides for downregulation of RUBCN gene expression (iRUBCN#1; iRUBCN#2) (Invitrogen; RUBNHSS145271, RUBNHSS145272) or ATG7 gene (iATG7#1, iATG7#2) (Invitrogen; ATG7HSS116184, ATG7HSS173705), plated at 250,000 cells/well in 6-well plates and infected with the hMpxV/Italy/un-INMI-Pt2/2022 isolate using a MOI of 0.5 TCID_50_/cell, as described above. An oligo not exerting silencing action on Rubicon or ATG7 was used as a control (iCtr; Invitrogen, GC medium 462001). At 0, 24, and 48 h.p.i. supernatants and cells from MPXV-infected cells silenced with iRUBCN#1 iRUBCN#2 and iCtr were harvested after the E64d/Pep.A treatment.

### Antibodies and Immunoblotting assays

The primary antibodies used in this study were rabbit anti-LC3 (Cell Signaling Technology, 2775), mouse anti SQSTM1/p62 (MBL, PM045), rabbit anti-L1R (Sino Biological, 40889-T62), mouse anti-Actin (Santa Cruz Biotecnology, sc-47778), mouse anti Tubulin (Santa Cruz Biotecnology, sc-32293), rabbit anti-Rubicon (Cell Signaling Technology, 8465), mouse anti-HSP90 (Santa Cruz Biotecnology, sc-13119), rabbit anti-Ambra1 (Millipore, ABC131), mouse anti Beclin1 (Santa Cruz, E-8, sc-48341), rabbit anti-ULK1 (Cell Signaling Technology, 8054), rabbit anti-UVRAG (Sigma-Aldrich, U7508), rabbit anti-Optineurin (Abcam, ab23666), rabbit anti NDP52 (Proteintech 12229-1-AP) (rabbit anti-p21 (Cell Signaling Technology, 2947), rabbit anti-p53 (Santa Cruz Biotecnology, sc-126), rabbit anti-ATG7 (Abcam, ab133528), rabbit anti-ubiquitin Lys48-specific (Millipore, 05-1307), mouse anti-GAPDH (Calbiochem, CB1001).

Cells were lysed in RIPA buffer: 150 mM NaCl (Sigma-Aldrich, S7653), 1% NP-40 (Sigma-Aldrich, 56741), 0.5% deoxycholic acid (MP Biomedicals, 101496), 0.1% SDS (Sigma-Aldrich, L3771), 50 mM tris (pH 8.0) (Santa Cruz Biotechnology, sc-3715A), and 2 mM MgCl_2_ (Sigma-Aldrich, M8266), with protease and phosphatase inhibitors: Protease Inhibitor Cocktail plus (Sigma-Aldrich, P8340), 5 mM sodium fluoride (Sigma-Aldrich, S-7920), 0.5 mM sodium orthovanadate (Sigma-Aldrich, S6508), 1 mM sodium molybdate (Sigma-Aldrich, S-6646), 50 mM 2-chloroacetamide (Sigma-Aldrich, C0267), 2 mM 1,10-phenanthroline monohydrate (Sigma-Aldrich, 320056), and 0.5 mM phenylmethylsulfonyl fluoride (Sigma-Aldrich, P7626).

Protein total extracts were separated on SDS–PAGE gels and electroblotted onto nitrocellulose (Whatman Amersham, 10600041) or PVDF (Millipore, IPVH20200) membranes. Blots were incubated with primary antibodies in 5% nonfat dry milk (Biosigma, 711160) or bovine serum albumin (BSA) (Sigma-Aldrich, A9647) in PBS (Thermo Fisher Scientific, 18912-0149) plus 0.1% Tween-20 (Sigma-Aldrich, P1379) overnight at 4 °C. Detection was achieved using horseradish peroxidase-conjugated secondary antibodies (Jackson ImmunoResearch Laboratories anti-goat 705-036-147, anti-rabbit 711-036-152, and anti-mouse 715-036-150) and enhanced chemiluminescence (ECL; Millipore, Immobilon Classico WBLUC0500 and Immobilon Crescendo Western HRP substrate WBLUR0500). Signals were acquired using a ChemiDoc imaging system. All samples were run in triplicate. Original Western blot images used for figures are provided in the Supplemental Material.

### Viral infectivity

Viral infectivity was evaluated by a limiting dilution assay in supernatants of Calu-3-infected cells with hMpxV/Italy/un-INMI-Pt2/2022 isolate for 48 h using an MOI of 0.5 TCID_50_/cell, transfected either with iRUBCN#1 or iRUBCN#2 or iCtr. In more detail, supernatants derived from the three different conditions were serially diluted in MEM supplemented with 2% FBS and then added to Vero E6 cells in a 96-well plate. After 6 days of incubation at 37 °C and 5% CO_2_, the appearance of cytopathic effect (CPE) was observed by light microscope, and the viral titer for each condition was evaluated using the Reed and Muench method [35] and expressed as TCID_50_/mL.

### Detection of MPXV DNA by real-time PCR

DNA extraction was performed both from supernatants and cells as follows: 140 μL of supernatants underwent DNA extraction using the QIAamp Viral DNA Mini Kit (Qiagen, Hilden, Germany) and were eluted in 60 μL of Elution buffer. DNA extraction from cells was performed using the Quik-DNA Microprep Kit (Zymo Research Corporation, Irvine, CA, USA) and, after elution in 15 μL of Elution buffer, DNA concentration was measured by NanoDrop™ 2000/2000c Spectrophotometers (Thermo Scientific, Italy). The amplification was performed using the real-time PCR published by Li et al. [36], targeting the gene encoding the CrmB secreted TNF-alpha-receptor-like protein of the MPXV genome on the Rotor-GeneQ Real-Time cycler (Qiagen, Hilden, Germany). A standard, previously quantified by the Bio-Rad QX200 AutoDG Digital Droplet PCR system (Bio-Rad, Hercules, CA, USA) [37], was serially diluted to obtain a curve enabling the transformation of cycle threshold (Ct) values from real-time PCR into quantitative results (qPCR) expressed as copies/mL or copies/ng.

### Real-time polymerase chain reaction

RNA was extracted with Trizol reagent (Invitrogen, 15596018). Complementary DNA synthesis was generated from 2 μg of RNA using the reverse transcription kit (Promega, A3500) according to the manufacturer’s recommendations. Real-time polymerase chain reactions (PCRs) were performed with the Quiagen Rotor Gene Q cycler using the Maxima SYBR Green/ROX qPCR Master Mix (Thermo Fisher Scientific, K0253) according to the manufacturer’s instructions. Two point five microliters of 1:5 complementary deoxyribonucleic acid (cDNA) was used as a template, and cycling parameters were 95 °C for 10 min, followed by 40 cycles of 95 °C for 15 s, 60 °C for 30 s, and 72 °C for 30 s. Levels of RUBCN RNAs were normalized to the HSP90 level using the equation 2−ΔCt. Primer sets for all amplicons were designed using the PrimerQuest™ Tool by Integrated DNA Technologies (IDT). List of used primers:

RUBCN human forward: 5’-GGCTTCATCTGTGAGTTCTG-3’

RUBCN human reverse: 5’-AGCAGGCTTTATGGTAACAC -3’

HSP90 human forward: 5’-GGAGGATCTCCCTCTAAACA-3’

HSP90 human reverse: 5’-CTTCCGCCAGTTCAGTAAAG-3’

### Statistical analysis

All figures were graphed using GraphPad 9 (GraphPad Software, La Jolla, CA, USA). Statistical significance between the results obtained from cells treated and not treated with E64d/Pep.A has been obtained using two-way ANOVA. When comparing the differences observed when treating with the oligos, a two-way ANOVA test has been used to determine the statistical significance in terms of MPXV DNA copy number reduction, while an ordinary one-way ANOVA has been used for viral infectivity. In all cases, *p*-values < 0.05 were considered significant.

Statistical analysis of immunoblots was performed using an unpaired, two-tailed Student’s *t*-test in GraphPad 9. Values are shown as mean ± standard error of the mean (SEM) of at least three independent experiments. *P* < 0.05, *P* ≤ 0.01, *P* ≤ 0.001, *P* ≤ 0.0001 were marked respectively by *, **, ***, ****. Densitometric analysis of immunoblots was performed using the Image Lab software (Biorad).

## Supplementary information

Figure S1

Figure legend S1

Figure S2

Figure legend S2

Original_Blots_1

Original_Blots_2

Original_Blots_3

Original_Blots_4

Original_Blots_5

## Data Availability

The original contributions presented in the study are included in the article; further inquiries can be directed to the corresponding authors.
