# Supplementary figures and images for: The monkeypox virus suppresses autophagy by modulating Rubicon expression
Source: Cell Death Discov. 2025 Dec 23;12:68. doi: 10.1038/s41420-025-02920-z (PMC12847765; doi:10.1038/s41420-025-02920-z)

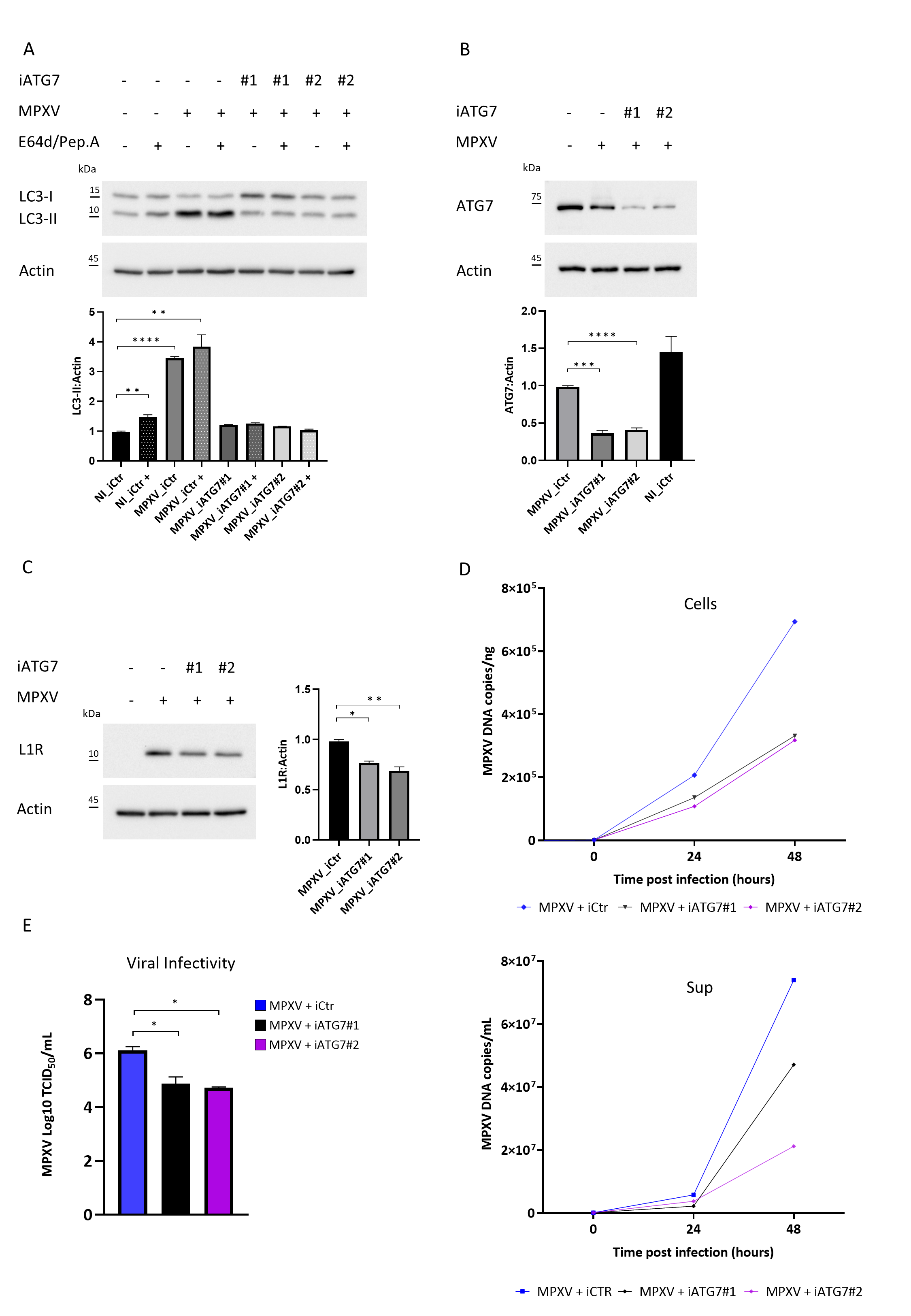

Supplement: Supplementary file 1 — Figure S1 [file 41420_2025_2920_MOESM1_ESM.tif]

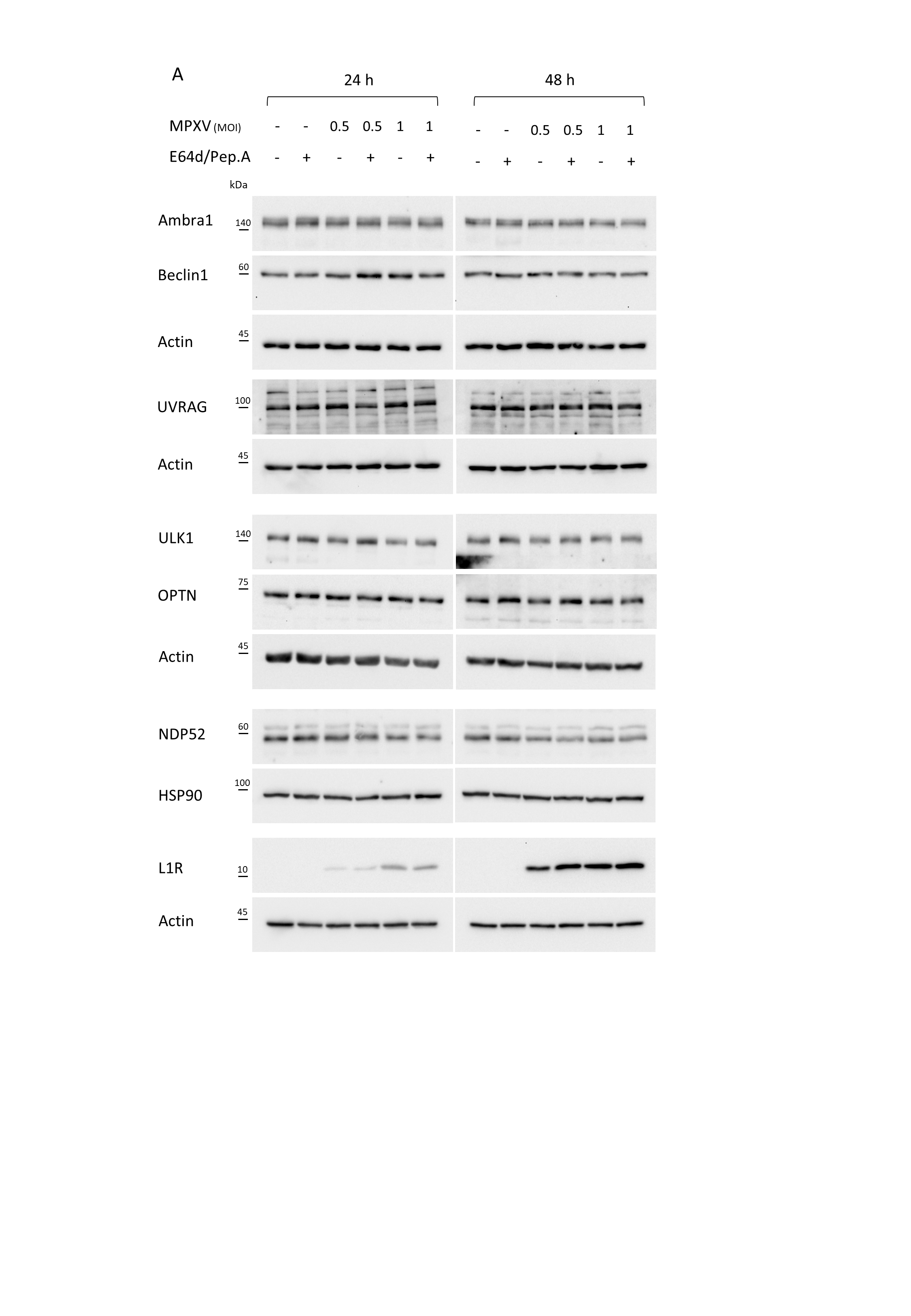

Supplement: Supplementary file 3 — Figure S2 [file 41420_2025_2920_MOESM3_ESM.tif]

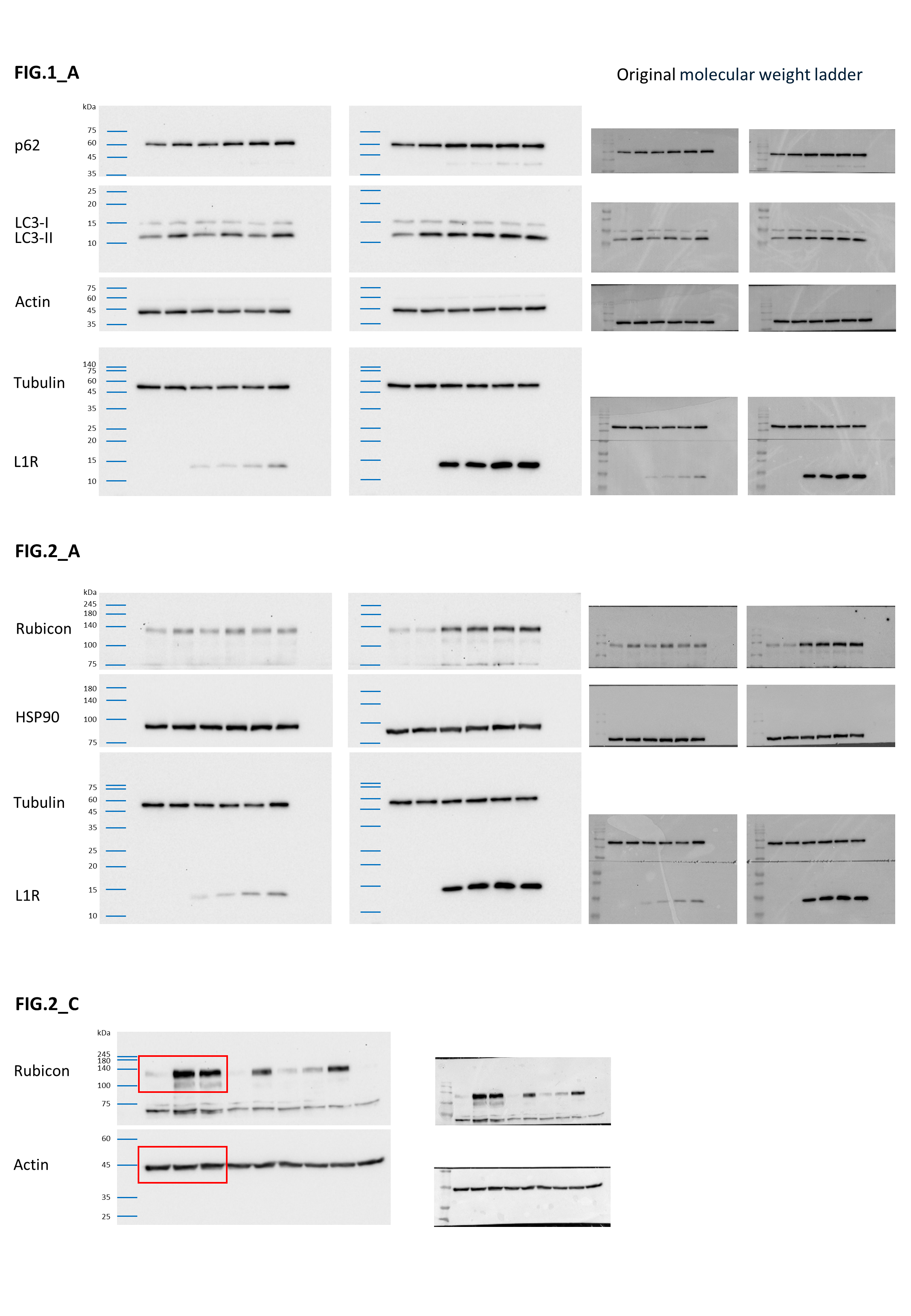

Supplement: Supplementary file 5 — Original_Blots_1 [file 41420_2025_2920_MOESM5_ESM.tif]

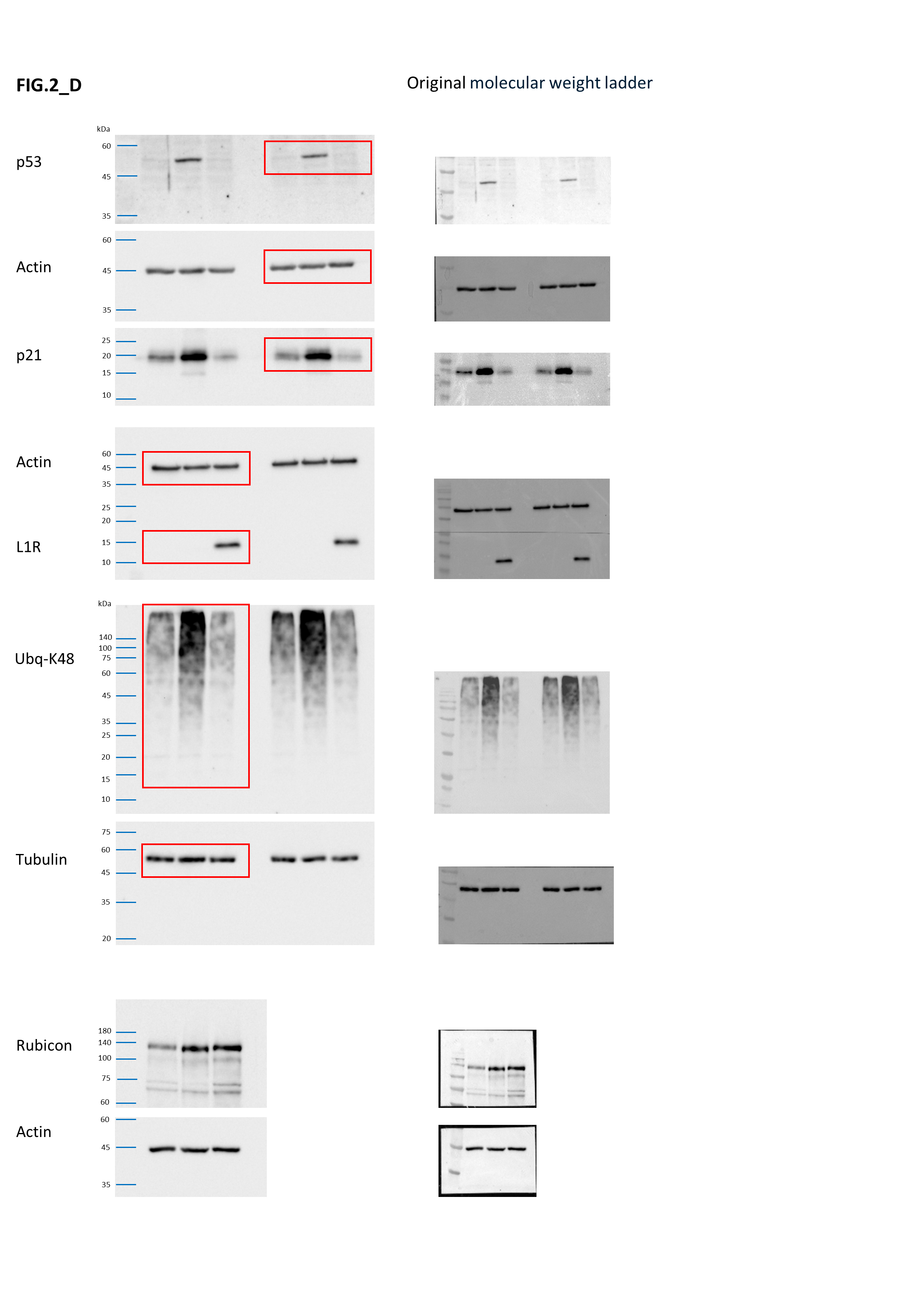

Supplement: Supplementary file 6 — Original_Blots_2 [file 41420_2025_2920_MOESM6_ESM.tif]

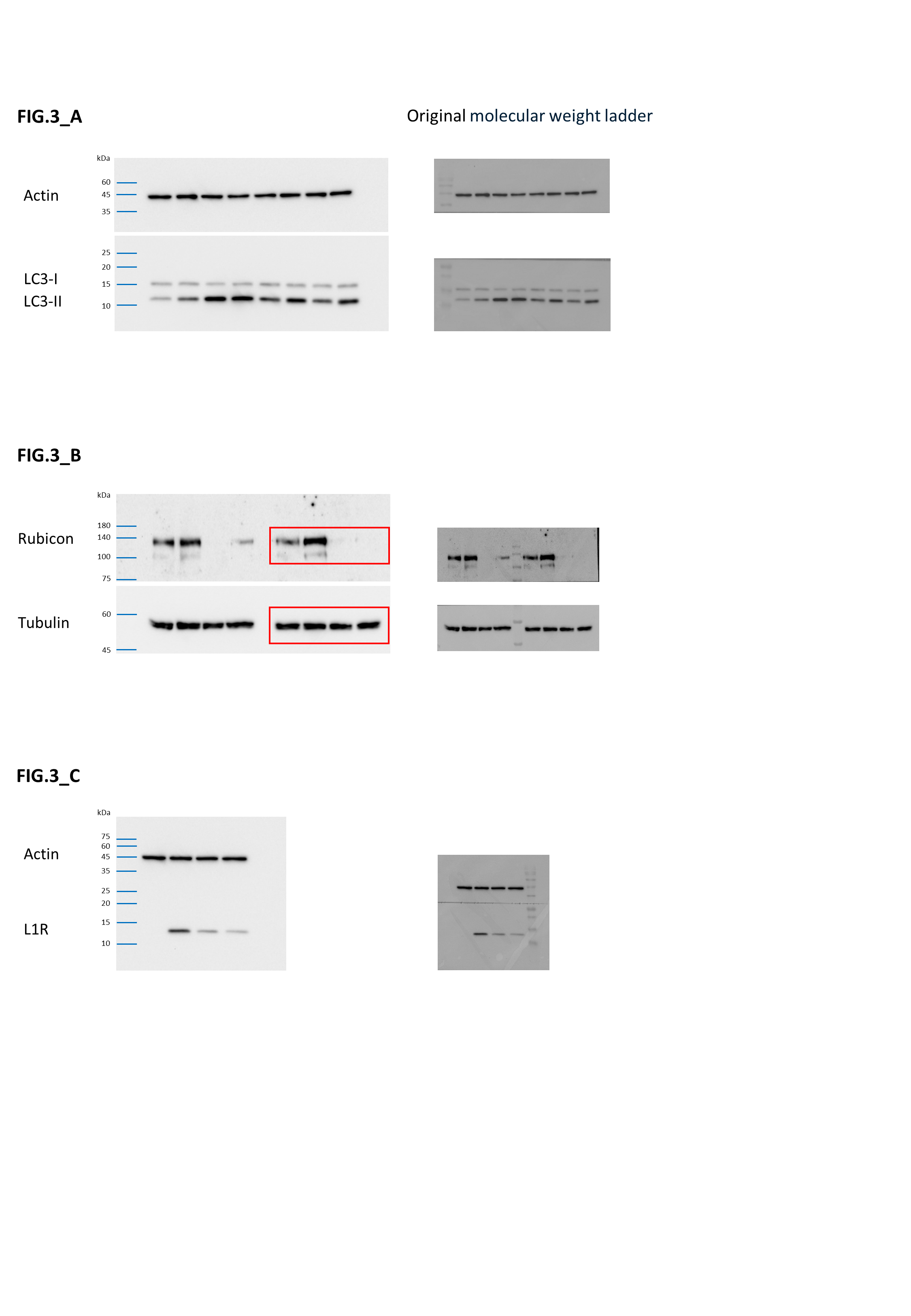

Supplement: Supplementary file 7 — Original_Blots_3 [file 41420_2025_2920_MOESM7_ESM.tif]

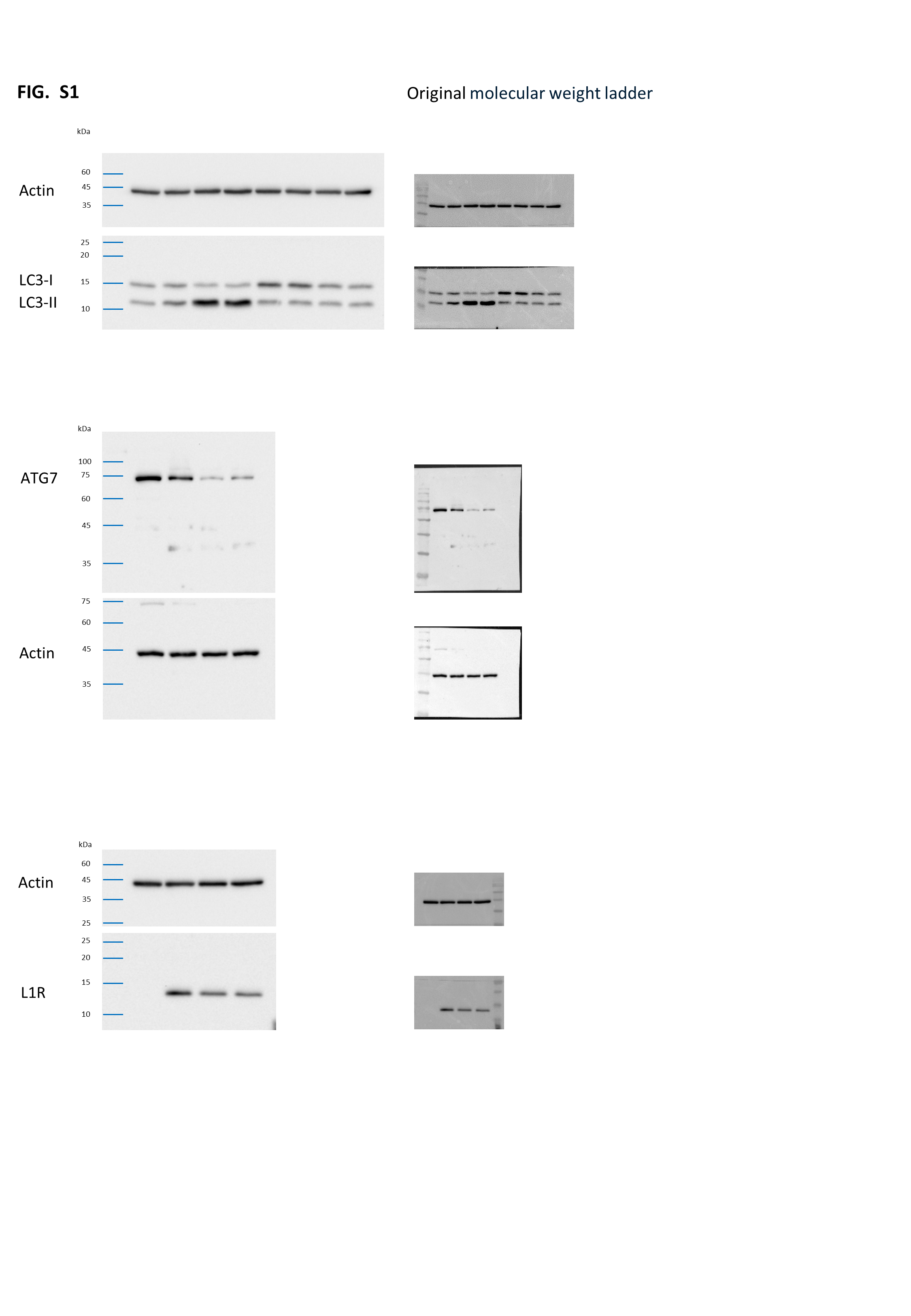

Supplement: Supplementary file 8 — Original_Blots_4 [file 41420_2025_2920_MOESM8_ESM.tif]

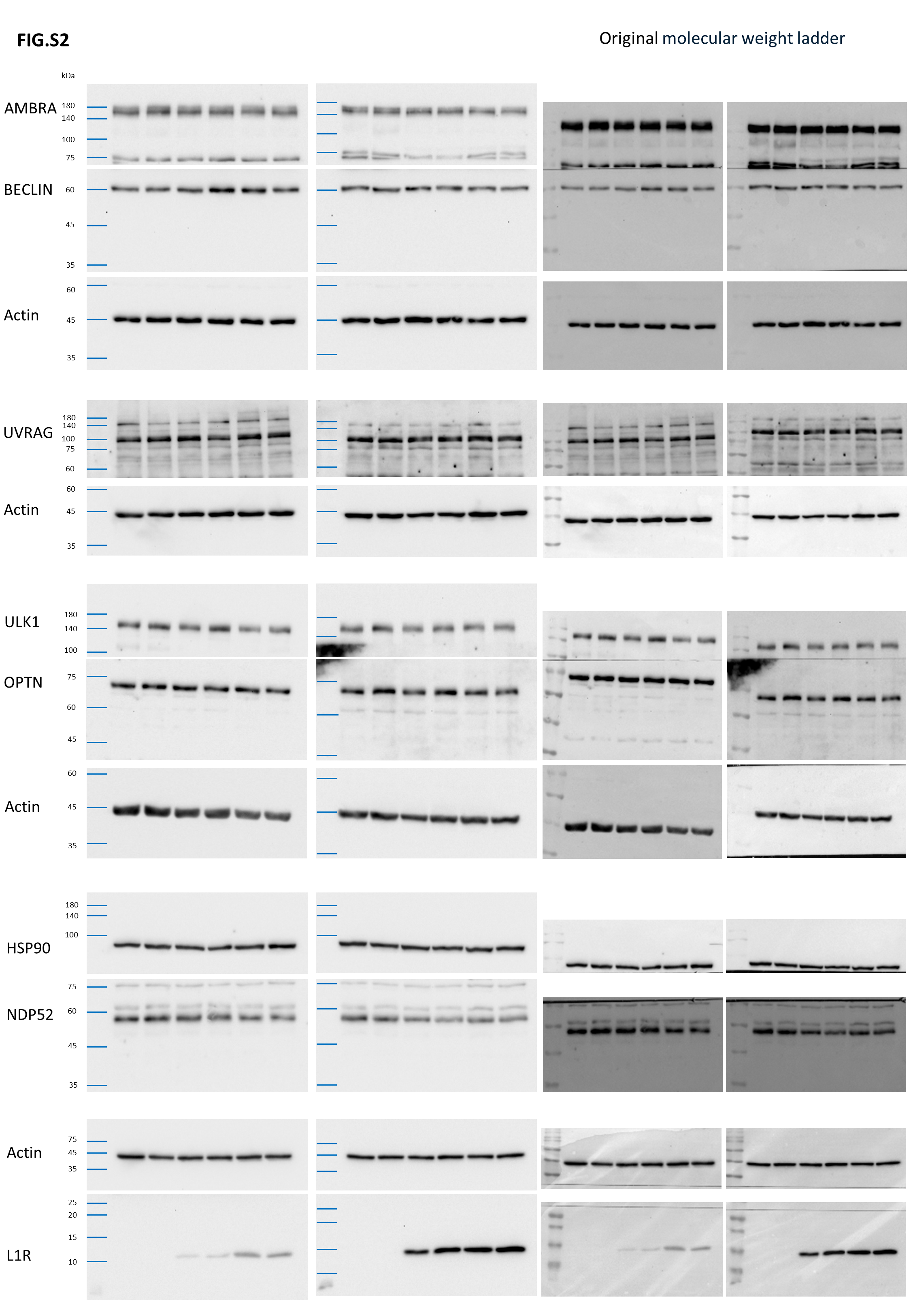

Supplement: Supplementary file 9 — Original_Blots_5 [file 41420_2025_2920_MOESM9_ESM.tif]
