# Supplementary material for: The monkeypox virus suppresses autophagy by modulating Rubicon expression
Source: Cell Death Discov. 2025 Dec 23;12:68. doi: 10.1038/s41420-025-02920-z (PMC12847765; doi:10.1038/s41420-025-02920-z)
Supplement: Supplementary file 2 — Figure legend S1 [file 41420_2025_2920_MOESM2_ESM.docx]

**Figure S1. ATG7 depletion impairs APs formation and affects MPXV replication**

Calu-3 cells silenced for ATG7 gene expression (iATG7#1, iATG7#2) or not (iCtr), were infected at MOI of 0.5 and cultured for 48 h. Two hours before lysis, cells were incubated with E64d/Pep.A as indicated (+). (**A**) LC3-II levels were analyzed to monitor autophagy flux by western blot. Actin was included as a loading control. The graph represent mean ± SEM of LC3-II:Actin values from three independent experiments. (**B**) ATG7 levels were analyzed by western blot. Actin was included as a loading control. The graphs represent means ± SEM of ATG7:Actin values from three independent experiments. (**C**) L1R levels were analyzed to control MPXV replication by western blot. Actin was included as a loading control. The graphs represent mean ± SEM of L1R:Actin values from three independent experiments. (**D**) Calu-3 cells were silenced for ATG7 gene expression: iATG7#1 (represented in black), iATG7#2 (represented in purple), or not (iCtr, represented in blue), infected at MOI of 0.5 and cultured for 48 h. Kinetic of viral yield inside the cells (Cells) (expressed as copies/ng) and in supernatants (Sup) (expressed as copies/mL) was quantified by qRT-PCR. (**E**) Viral titer was evaluated as Log TCID50/mL, in supernatants of Calu-3 infected cells with MPXV at MOI of 0.5 for 48h, and transfected either with iATG7#1 (represented in black) or iATG7#2 (represented in purple) and iCtr (represented in blue). Experiments were performed as three independent replicates; mean ± SEM are shown in the picture.
