# Supplementary material for: The monkeypox virus suppresses autophagy by modulating Rubicon expression
Source: Cell Death Discov. 2025 Dec 23;12:68. doi: 10.1038/s41420-025-02920-z (PMC12847765; doi:10.1038/s41420-025-02920-z)
Supplement: Supplementary file 4 — Figure legend S2 [file 41420_2025_2920_MOESM4_ESM.docx]

**Figure S2. Analysis of MPXV infection on autophagic regulator proteins**

(**A**) Calu-3 cells were infected with MPXV at MOI of 0.5 or 1 for 24 h and 48 h. Two hours before lysis, cells were incubated with E64d/Pep.A as indicated (+). Ambra1, Beclin1, UVRAG, ULK1, OPTN and NDP52 were analyzed by western blot. L1R levels were analyzed to verify MPXV replication. Actin and HSP90 were included as loading control. Experiments were performed as three independent replicates. No significant modulations were observed (data not shown).
